# Characteristics of Chinese Costal Cartilage and Costa Calcification Using Dual-Energy Computed Tomography Imaging

**DOI:** 10.1038/s41598-017-02859-x

**Published:** 2017-06-07

**Authors:** Shanyong Zhang, Jinze Zhen, Huiping Li, Shoufu Sun, Huawei Wu, Pei Shen, Zengai Chen, Chi Yang

**Affiliations:** 1 Department of Oral Surgery, Ninth People’s Hospital, College of Stomatology, Shanghai Jiao Tong University School of Medicine, Shanghai Key Laboratory of Stomatology & Shanghai Research Institute of Stomatology, Shanghai, People’s Republic of China; 2 Department of Radiology, Renji Hospital, Shanghai Jiao Tong University School of Medicine, 160 Pujian Road, Shanghai, 200127 People’s Republic of China

## Abstract

To assess characteristics of Chinese costal cartilage and costa calcification using Dual-Energy computed tomography(DECT). 154 patients who underwent chest DECT scanning were included in our study. They were divided into following groups: less than 30 years old, 31–40 years old, 41–50 years old, 51–60 years old and over 60 years old. The sixth, seventh and eighth costal cartilages and costas were evaluated. Calcification patterns of cartilage were classified as central(C), peripheral(P), mixed(M) and no calcification(N) types. Calcification degree of cartilage was distinguished as 1(0–25%), 2(26–50%) and 3(>50%). CT value, calcium and water concentrations were measured in costal cartilage, cortical or cancellous bone respectively. An increasing C pattern of cartilage was displayed in females, while P type preferred in males as age increased. Calcification degree generally changed from 1 to 2 or 3 in females. CT value and calcium concentration of cartilage went through a gradual rising course and peaked in their 40–50 years, while those two indices of cancellous bone decreased gradually since their 50 years in females. The findings suggest a gradual calcification of the costal cartilage took place before 40–50 years old and a sharp bone loss of the costa happened after 40–50 years old in females.

## Introduction

Costal Cartilage or costa grafts are widely used in the clinical fields because of their good biological compatibility, no rejection and easy survival characters compared with other materials. Autologous costal cartilages can be grafted and carved into the shapes of nasal dorsum or auricula, which are quite common in rhinoplasty and ear reconstruction^1–3^. Besides costochondral grafts are also considered to be ideal donor tissues for temporomandibular joint reconstruction as there is a low incidence of complications at the donor site, good adaptability to the mandibular ramus and incorporation of the head of cartilage tissue. These advatanges promote the morphological and functional adaptations to the temporomandibular joint^4, 5^.

On the other hand, the costal cartilage or costa grafts have the inherent limitations because of their age-related changes. Calcification in both costal cartilage and costa will change with the increase of age. These histological changes can results in the difference of biomechanical properties, which can affect the treatment effects. So-Eun Han^6^ reported that calcification and the associated stiffness of costal cartilage with increasing age were important factors that leaded to unexpected absorption and poor surgical results. Wofford^7^ believed that costochondral costal grafts were better used in children and adolescents because of the poor quality of costa in the elderly. Thus, it is an important problem how to evaluate the morphological and histological characteristics before surgery and calculate the degree of calcification of costal cartilage or costa.

Although many studies reported that the calcification of costal cartilage and costa played a key role in the outcome of grafted surgery, only a few studies have provided information on the characteristics of costal cartilage calcification and there are no studies about the calcification of costa^8, 9^. Moreover most studies only have displayed the degree of costal cartilage calcification but with no accurate value about the calcium content. This is because to date all the studies used computed tomographic (CT) scans to analysis the costal cartilage. Whether there is a better instrument to evaluate characteristics of both costal cartilage and costa and provide more information on them can contribute to make better decision before the surgery. In recent years, Dual-Energy CT(DECT) plays an increasing role in the clinical fields due to its quantitative density measurement and monochromatic spectral images. This is because DE imaging allows tissue characterization and functional analysis at energy levels ranging from 40 to 140 keV compared with conventional CT^10^. The aim of our study was to assess characteristics of Chinese costal cartilage and costa calcification using DECT. In general, the sixth, seventh, or eighth costal cartilage and costa are the most often harvested in the clinic, so in the present study we only assessed these three costal cartilage and costa.

## Results

### Calcification Patterns of Costal Cartilage

**Figure 1:**
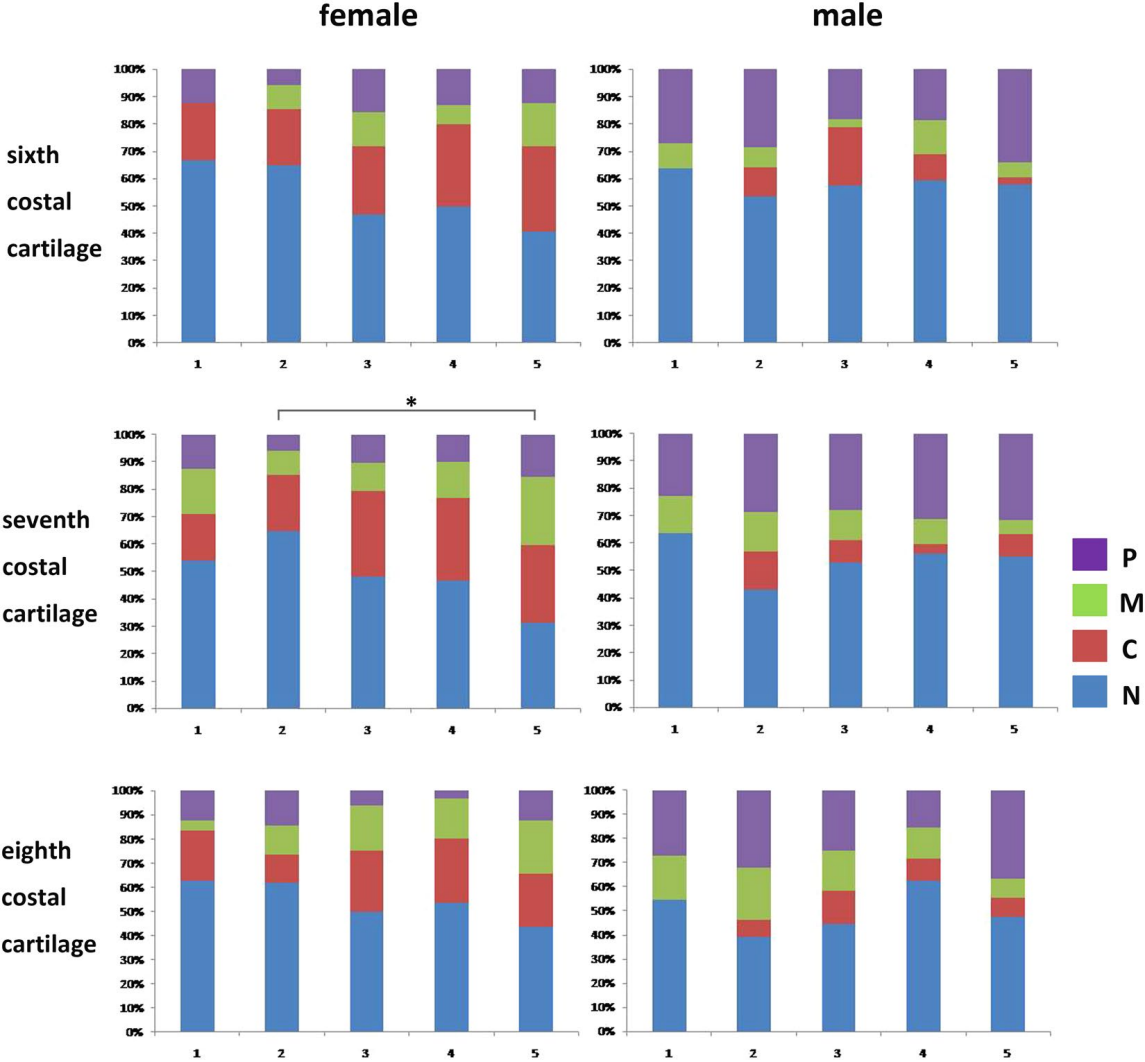
The characteristics of costal cartilage calcification patterns in different age groups. A decreasing N calcification type, as well as an increasing C pattern, was displayed in the female patients with age increased. P calcification type appeared more frequently in male patients in all the three costal cartilages. *P < 0.05.

In both female and male patients, the costal cartilages predominantly showed N type, whereas the calcification patterns changed with the increase of age (Fig. 1). Compared with male patients, a decreasing N calcification type, as well as an increasing C pattern, was displayed in the female patients as their age increased, especially in the sixth and seventh costal cartilages. While P calcification type appeared more frequently in male patients in all the three costal cartilages.

### Calcification Degree of Costal Cartilage

**Figure 2:**
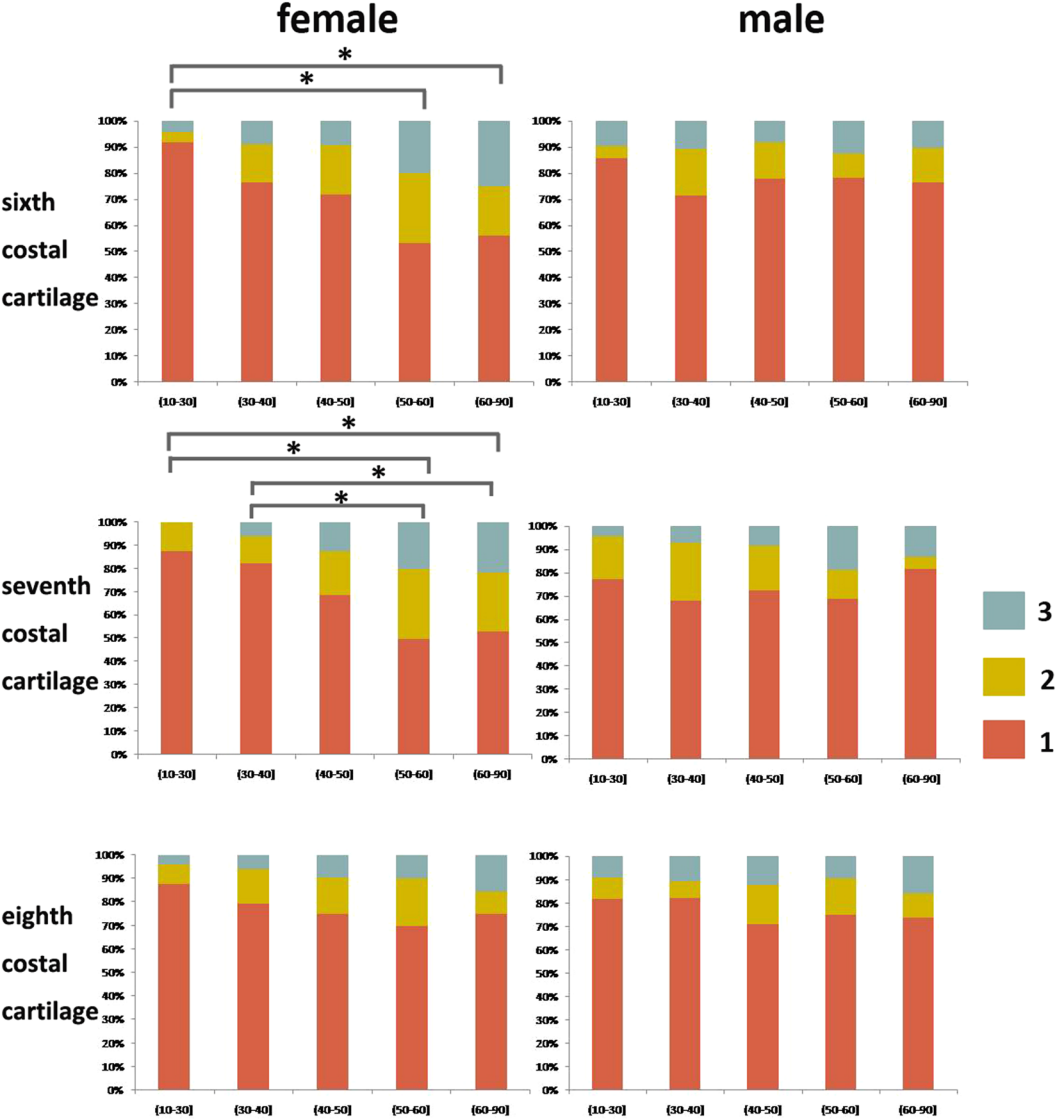
he calcification degree of costal cartilage in different age groups. A gradual increased calcification degree of costal cartilage happened in female patients. However significant differences were only found in the sixth and seventh costal cartilage in female patients. *P < 0.05.

As a whole, female patients showed more frequent calcification than male patients. The calcification degree generally changed from degree 1 to degree 2 or 3 with age increased in female patients, especially in the sixth and seventh costal cartilages (Fig. 2). However this trend of costal cartilage calcification in male patients was not as apparent as in female patients. A significant difference was only found in female patients in the sixth and seventh costal cartilage (P < 0.05). In both sexes, the eighth costal cartilage had the lowest degree of calcification.

### CT value, calcium concentration and water concentration of Costal Cartilage and costa

**Figure 3:**
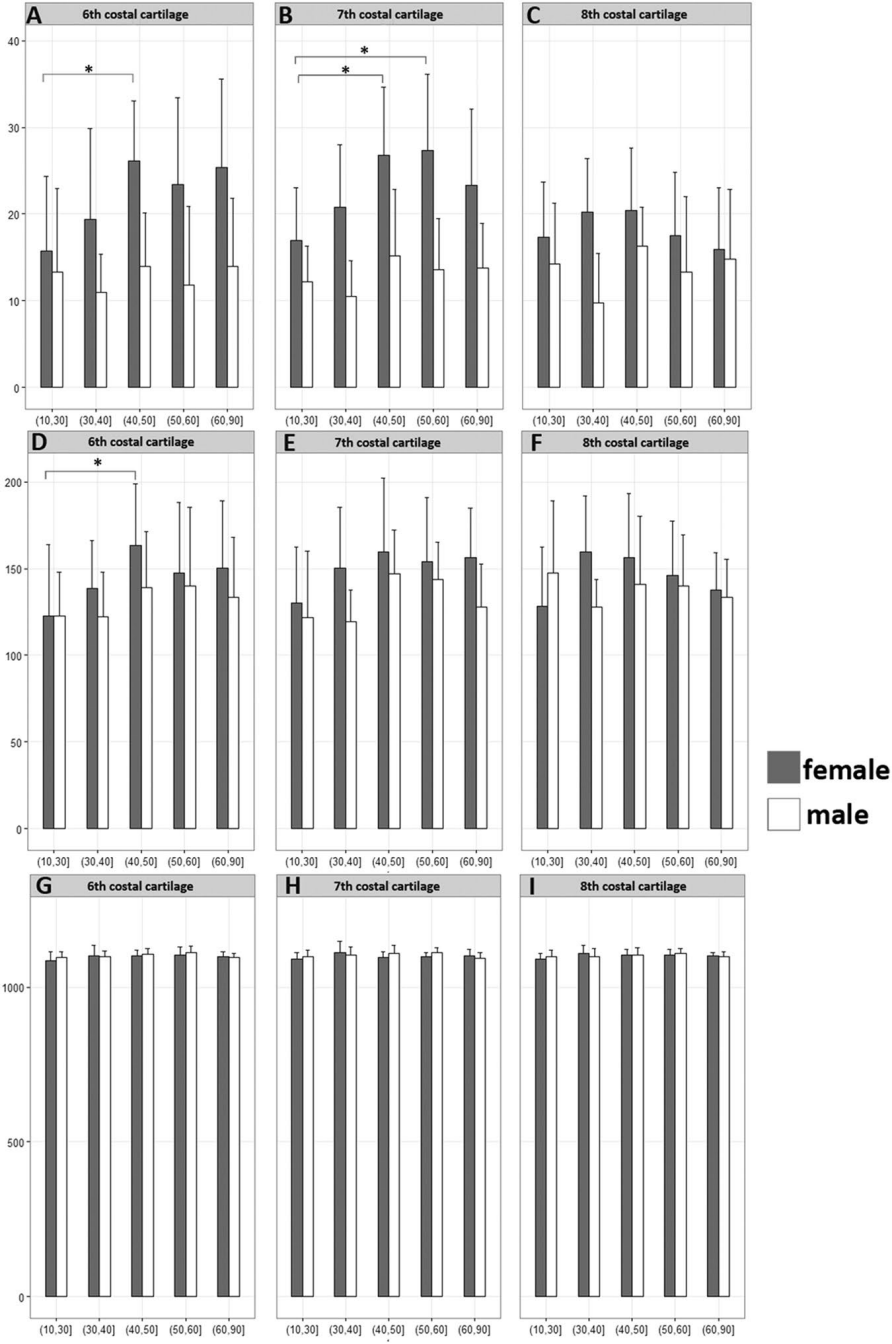
The measurement results of costal cartilage. (**A**,**B**,**C**) The calcium concentration of costal cartilage in different groups. A gradual increase of calcium concentration was found before 50 years old groups in female patients and a slight decrease after 50 years old. (**D**,**E**,**F**) The CT values of costal cartilages in different age groups. The CT values of costal cartilages went through a gradual rising before 50 years old in female patients, which were in accordance with the tendency of calcium concentration. (**G**,**H**,**I**) The water concentration of costal cartilage in different groups. The water concentration both in female and male patients was almost the same in all age groups. *P < 0.05.

As a whole, obvious variations in different age groups were more frequently observed in female patients than in male patients. In the costal cartilage, the CT value and calcium concentration went through a gradual rising course and peaked in the 40–50 years old in female patients. While in the male patients, there were no obvious changes in the CT value and calcium concentration in all age groups. Significant differences were only found in these female groups in the sixth and seventh costal cartilage (P < 0.05) (Fig. 3). The water concentration both in female and male patients was almost the same in all age groups.

**Figure 4:**
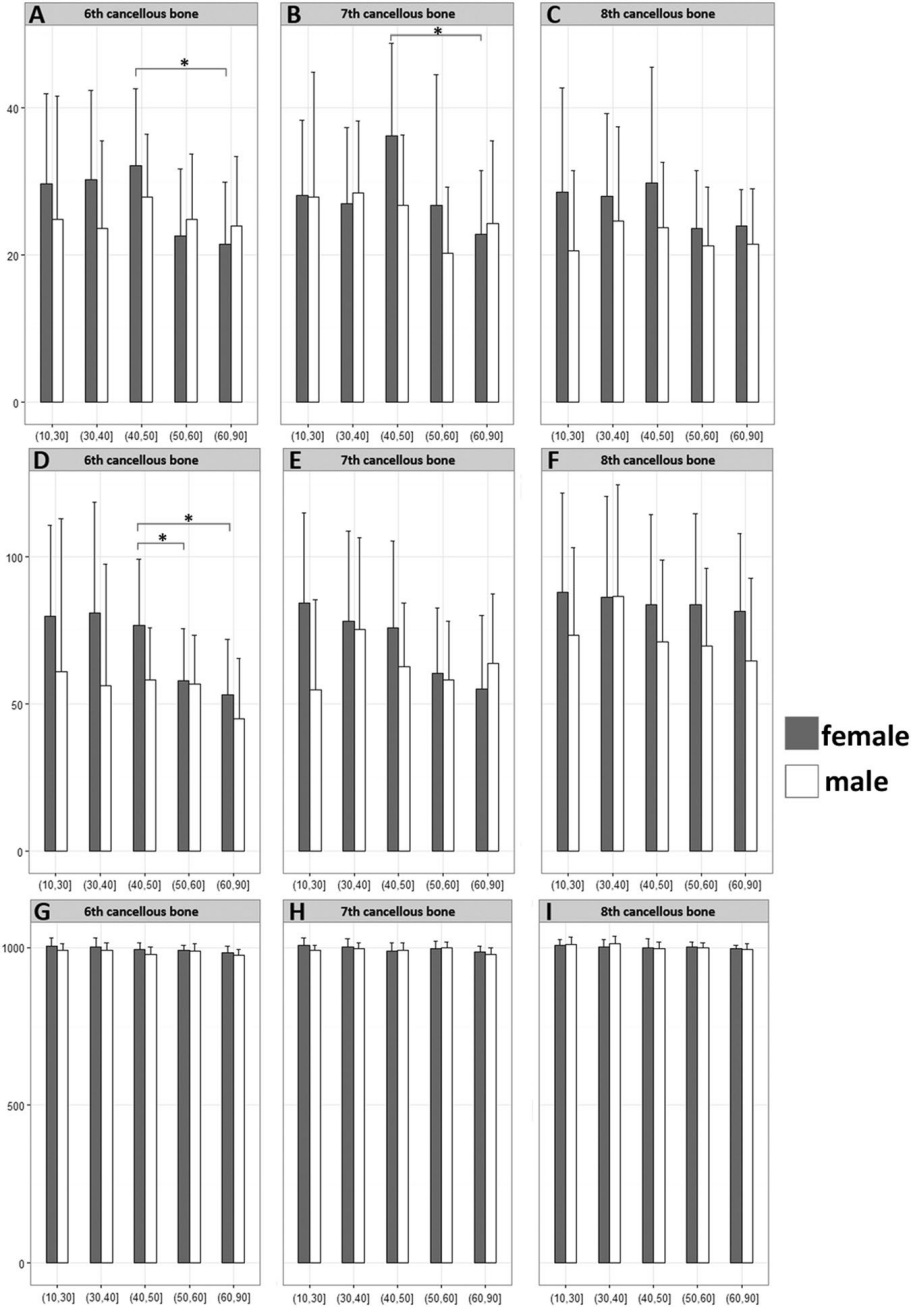
The measurement results of costal cancellous bone. (**A**,**B**,**C**) The calcium concentration of costal cancellous bone in different groups. (**D**,**E**,**F**) The CT values of costal cancellous bone in different age groups. the CT value and calcium concentration of costal cancellous bone decreased gradually since the 50 years old in female patients, whereas this two values were similar in male patients in all groups. (**G**,**H**,**I**) The water concentration of costal cancellous bone in different groups. *P < 0.05.

**Figure 5:**
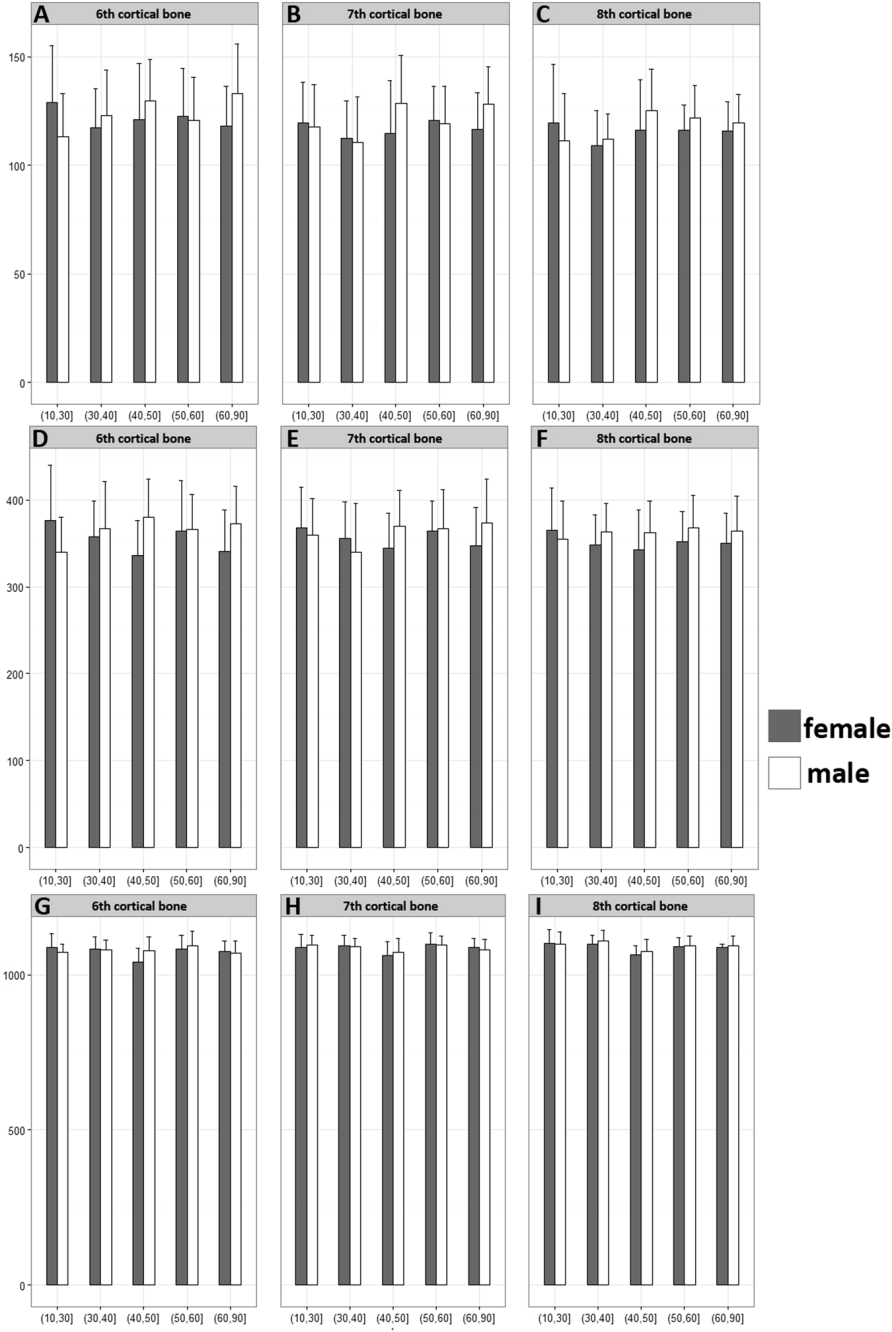
The measurement results of costal cortical bone. (**A**,**B**,**C**) The calcium concentration of costal cortical bone in different groups. (**D**,**E**,**F**) The CT values of costal cortical bone in different age groups. (**G**,**H**,**I**) The water concentration of costal cortical bone in different groups. No significant changes were found in all these three values of costal cortical bone.

Compared with costal cortical bone, the costal cancellous bone have a more significant difference with age. In the sixth and seventh costas, the CT value and calcium concentration of costal cancellous bone decreased gradually since the 50 years old in female patients, whereas this two values of the two costal cancellous bone were similar in male patients in all groups (Fig. 4). The CT value and calcium concentration of the eighth costal cancellous bone did not change so obviously compared with the other two costas in different age groups. However the absolute CT value of the eighth costa was higher than another two costal cancellous bones. The cortical bone of the three costa shared a similar CT value and calcium concentration (Fig. 5). The water concentration of the cortical bone was higher than that of cancellous bone although there were almost no significant changes in different age or sex groups.

## Discussion

Costal Cartilage and costa are ideal donor tissues in the reparative and reconstructive surgery fields. However the qualities of costal cartilage and costa play a critical role in the treatment effects. Although many studies reported that costal cartilage increased with age, and had a relationship with sex^11, 12^, no reports elaborated the exact relationship between costal cartilage calcification and age or sex. Besides, almost all the studies focused on the cartilage calcification and there were no references paying attention to the costa age related changes. As is known to us, osteoporosis is proved in menopausal women, whether this process could affect costa and what is the difference between male and female in different age groups? As a result, we have measured the calcification of both costal cartilage and costa and compared the difference with age in the present study.

Calcification of costal cartilage often draws much attention of scholars and doctors. Bozzato A^13^ once used ultrasonography to evaluate the calcification patterns of costal cartilage. His reports showed a trend toward a C pattern for women and a P pattern among men. Similar with Bozzato’s study, our results also showed a tendency to the C pattern in female and P pattern preferred in male. Moreover, the C pattern calcification slightly increased in female and P pattern in male with age growing. In accordance with the calcification patterns, the calcification degree gradually increased with age in female patients, especially in the sixth and seventh costal cartilages. The CT measurement and Ca volume also gradually rose and peaked in 40–50 years old female patients, which suggested more severe calcification in this group, compared with the younger groups. Combined the results of costal cartilage, the female showed more significant age related changes, while the male with no apparent changes in different age groups. Besides, the sixth and seventh costal cartilages appeared to be more susceptible to age and sex than the eighth costal cartilage.

In contrast with the cartilage, the calcium volume in costal cancellous bone showed a sharp decline since the 50 years old in female patients, which leaded to decrease in CT measurement. However the costal cortical bone almost shared a similar calcium volume and CT measurements in different age groups. This suggested that female underwent a significant bone loss around the 50 years old and the major change occurred in the costal cancellous bone. There are many life events contributing to the bone loss and one of the most important event is menopause in the female patients^14, 15^. Although bone loss in menopausal women has been conformed in many studies, there were no reports discussing the costa bone calcium in this special population^16, 17^. So our study is the first time to provide information on the costa calcium loss as age increased in female patients.

In the clinical work, the different calcification pattern and degree of costal cartilage and costa can result different graft results. For example, though P pattern calcification of the costal cartilage may have some difficulties for grafts, the central portion can be used safely. Whereas, a C or M calcification pattern make the cartilage difficult to graft because of the poor clinical outcomes. On the other hand, as bone loss of the costa increased in the aged female patients, the graft survival rate reduced accordingly, which makes the costa no longer fit for grafts.

## Conclusion

In summary, this study is the first report about the accurate calcification volume of costal cartilage and costa using DECT. The results showed an age and sex related calcification changes of the costal cartilage and costa. A gradual calcification of the costal cartilage took place before 40–50 years old and a sharp bone loss of the costa happened after 40–50 years old in females. However the males did not undergo significant calcification changes of costal cartilage and costa during their whole lives.

## Materials and Methods

### Ethics Statement

This study followed the tenets of the Declaration of Helsinki for research involving human subjects, informed consent was obtained from all participants, and the study was critically reviewed and approved by the institutional review board of Shanghai Jiao Tong University School of Medicine.

### Patients

A total of 154 patients who underwent chest DECT scanning over a two-years period (from January 2013 to December 2014) at the Renji People’s Hospital, Shanghai Jiao Tong University, School of Medicine, were included in our study. The inclusion criteria were as follows: ① without thoracodynia; ② without Diseases of the chest wall; ③ without histories of chest trauma, radiotherapy or surgery; ④ without history of bone or cartilage tumor or systemic diseases. These patients were consist of 76 female and 78 male with a mean age of 47 ± 15.8 years old (range from 19 to 82). The patients were divided into following groups according to their age: less than 30 years old (23 cases), 31–40 years old (31 cases), 41–50 years old (34 cases), 51–60 years old (31 cases) and more than 60 years old (35 cases)(Table 1). This study was conducted in accordance with the Ethics Committee of Shanghai JiaoTong University School of Medicine.

**Table 1:** Demographics of the groups in this study.

Group	Age Mean(SD)	Sex	
		female	male
(10, 30]	25.1(3.5)	12	11
(30, 40]	35.1(2.9)	17	14
(40, 50]	45.6(2.7)	16	18
(50, 60]	56.0(2.6)	15	16
(60, 90]	69.6(6.9)	16	19

### CT examination

Non-enhanced CT examinations were conducted using Discovery CT750HD (HDCT) (GE Healthcare, Wisconsin, USA) scanner. A scout scan was taken first to plan for the spiral acquisition, which included the whole chest from the first costa to the twelfth costa. After scout CT scanning, non-enhanced CT was obtained with spectral imaging mode, with fast tube voltage switching between 80 and 140 kVp on adjacent views during a single rotation^18^. The default 70 keV monochromatic images were used for quantifying CT value. The CT parameters were as follows: tube rotation time, 0.6 seconds; collimation, 1.25 mm; tube current, 600 mA; pitch, 1.375; slice thickness and interval for axial images, 5 mm/5 mm; field-of-view, 500 mm. The rotation time of 0.6 seconds was shared by the 80 kVp and 140 kVp with uneven distribution to balance the signals. GSI viewer software package, which can automatically calculate the CT values and calcium (water) concentration for the costal cartilage and costa, was used to process projection data and review images^19^. Two types of images were reconstructed from the single spectral CT acquisition for analysis: water- and calcification-based material decomposition images and a set of monochromatic images obtained at energies ranging from 40 to 140 keV^20^. The monochromatic images were used for quantifying CT value, and the calcification-based material decomposition images were used for quantifying calcification concentration.

### Calcification Evaluation of the Costal Cartilage and Costa

**Figure 6:**
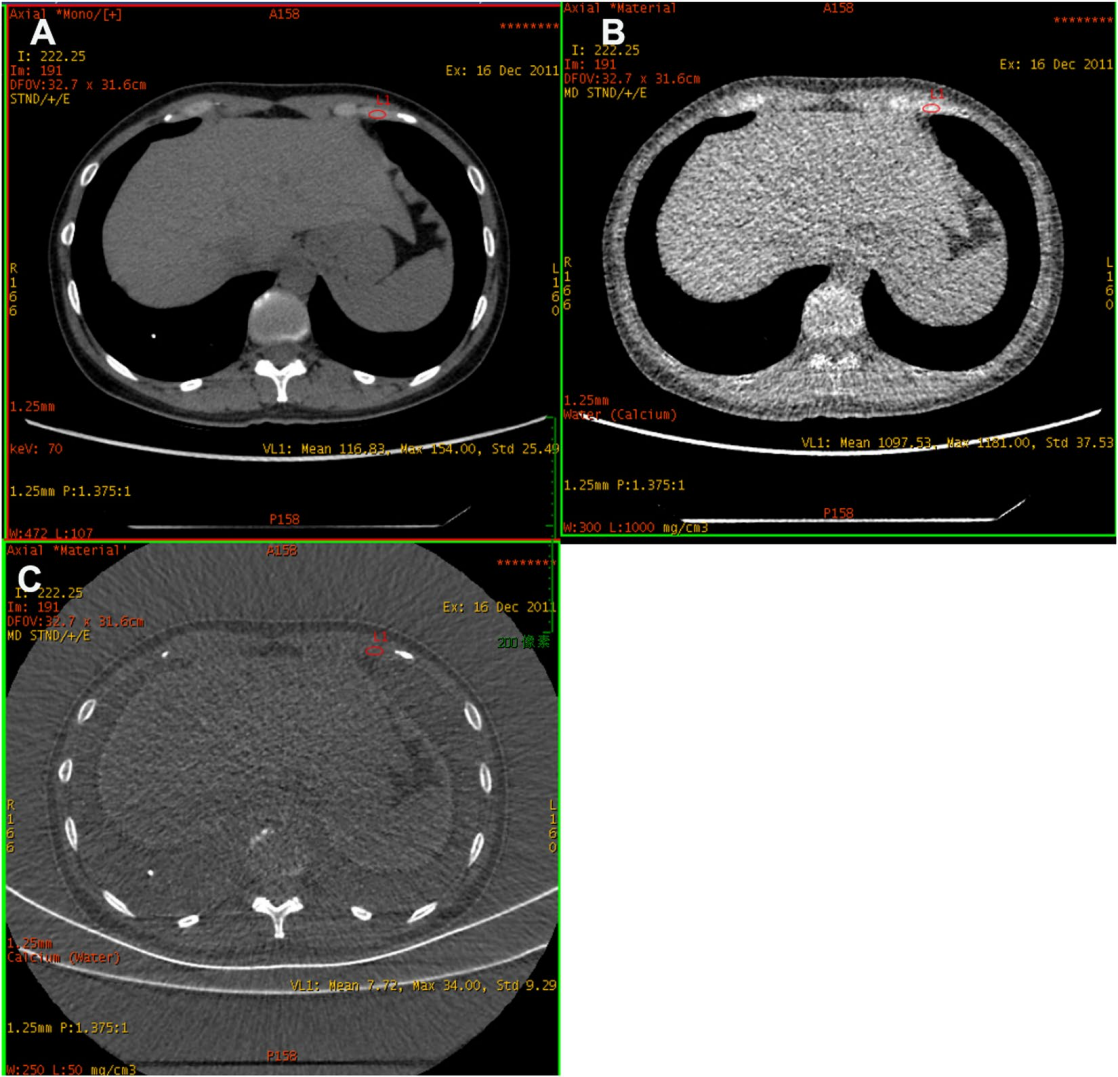
The evaluation of costal cartilage using DECT. (**A**) The CT value of costal cartilage. (**B**) The water concentration of costal cartilage. (**C**) The calcium concentration of costal cartilage.

**Figure 7:**
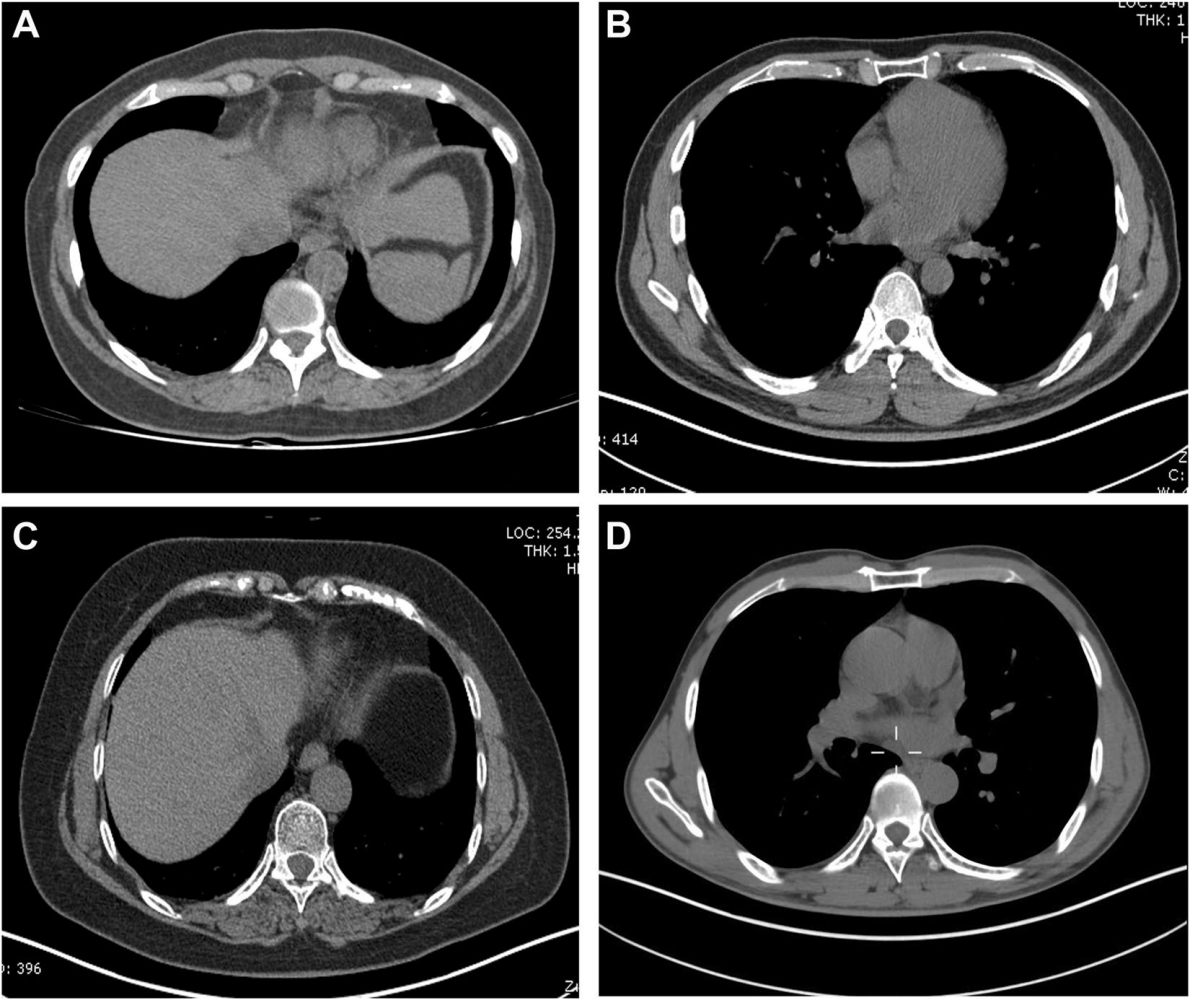
Calcification patterns of costal cartilage. Calcification patterns of costal cartilage were divided into the following types: (**A**) central, (**B**) peripheral, (**C**) mixed and (**D**) no calcification.

The sixth, seventh and eighth costal cartilage and costa were evaluated in our study. Regions of interest (ROIs) were selected as follows: planes 2 mm, 4 mm, 6 mm, 8 mm and 10 mm from the junction of costicartilage and costa. In each measuring plane, eight directions were equally extended from the center of costicartilage or costa respectively. Four marked points in every direction were measured for three times to acquire the average value. The CT value, calcium concentration and water concentration were measured in costal cartilage, costal cortical bone and cancellous bone respectively (Fig. 6). The calcification patterns of costal cartilage were classified as central (C), peripheral(P), mixed(M) and no calcification(N) types according to Navani’s and Sanders’s studies^21, 22^ (Fig. 7). The calcification degree of costal cartilage was distinguished as the following 3 grades: 1 (0–25%), 2 (26–50%), 3 (>50%). The degree of calcification was measured according to the ratio between the maximum calcification area and the total area in each measurement plane. All the measurements were carried out by two highly experienced technicians. All the technologists had performed radiology for more than 10 years. The two radiologists calculated the value separately. When their measurements differed too much, a third specialist who had more experience with radiology evaluated the images. The mean value of measurements by the two radiologists was used to calculate the final calcification degree of costal cartilage and costa.

### Statistical Analysis

Statistical analysis was performed with R Core Team (2015). (R: A language and environment for statistical computing; R Foundation for Statistical Computing, Vienna, Austria). Statistically significant differences in mean values of the CT value, calcium concentration and water concentration were tested by one-way ANOVA and the TukeyHSD for comparisons of multiple groups. The χ^2^ tests or Fisher exact test were used for calcification pattern and calcification degree. The differences were considered significant when p < 0.05.
